# Supplementary material for: Body Mass Index and Mortality, Recurrence and Readmission after Myocardial Infarction: Systematic Review and Meta-Analysis
Source: J Clin Med. 2022 May 5;11(9):2581. doi: 10.3390/jcm11092581 (PMC9104293; doi:10.3390/jcm11092581)
Supplement: Supplementary file 1 [file jcm-11-02581-s001.zip › jcm-1689346-supplementary.pdf]

SUPPLEMENTARY FILE

Supplementary Table S1: Search strategy used in Medline (Ovid). Similar search terms were used in EMBASE (Ovid) and Web of Science and the search strategy was revised to suit each database.

1. BMI.mp
2. body fat distribution/ or body mass index/ or body size/ or body weight/ or waist circumference/ or skinfold thickness/ or waist-hip ratio/
3. weight change or weight loss
4. body weight changes/ or weight loss/ or thinness/
5. adiposity.mp.
6. adiposity/ or body weight/ or waist circumference/ or skinfold thickness/ or waist-hip ratio/ 7. Creatinine.mp.
8. *Creatinine/bl, ur [Blood, Urine]
9. Malnutrition.mp.
10. malnutrition/ or deficiency diseases/ or magnesium deficiency/ or potassium deficiency/ or protein deficiency/ or protein-energy malnutrition/
11. Low albumin or Low prealbumin or Low transferrin.
12. Prealbumin/bl [Blood]
13. *Transferrin/bl [Blood]
14. *Serum Albumin/bl [Blood]
15. (hydrat* or dehydrat*).mp.
16. dehydration/ or hypercalcemia/ or hyperkalemia/ or hypernatremia/ or hypocalcemia/ or hypokalemia/ or hyponatremia/
17. 1 or 2 or 3 or 4 or 5 or 6 or 7 or 8 or 9 or 10 or 11 or 12 or 13 or 14 or 15 or 16
18. cohort.mp.
19. cohort studies/ or follow-up studies/ or prospective studies/
20. Myocardial Infarction.mp.
21. myocardial infarction/ or anterior wall myocardial infarction/ or inferior wall myocardial infarction/

22. stroke.mp.

23. Brain ischemia/ or hypoxia-ischemia, brain/ or "intracranial embolism and thrombosis"/ or intracranial embolism/ or intracranial thrombosis/ or intracranial hemorrhages/ or cerebral haemorrhage/ or intracranial haemorrhage, hypertensive/ or stroke/

24. Transient ischemic attack.mp.

25. brain ischemia/ or ischemic attack, transient/

26. 20 or 21 or 22 or 23 or 24 or 25

27. 18 or 19

28. 17 and 26 and 27

Supplementary Table S2: Data extraction form

Cohort Data Extraction Form and Validity Tool

Article Information

**Author**	
**Journal**	
**Year**	
**Study title**	

Study Characteristics

**Country of origin**		
**Language**		
**Dates for Cohort Enrolment**	From:	To:
**Duration of study follow up**		
**Drop out**		
**Reason for dropouts**		

Subject Characteristics

**Total population selected**		
**Total population included**		
**Mean age**		
**Males/Females**	Males:	Females:
**Age range**		
**Inclusion criteria**		

Exposure: Which of the following Exposures assessed in the study?

a. Myocardial infarction b. Transient ischemic attack c. Stroke

Define Malnutrition: How is malnutrition defined in this study? (If applicable)

Nutrition Markers: Which Nutrition markers/assessment tools were measured/evaluated in this study?

Indicator	Tick below if used	Cut off values defined as malnutrition	Number below off values	Number malnourished
BMI				
Weight				
Malnutrition tool				
Mid Arm Circumference				
Triceps skinfold				
Serum Albumin				
Serum Creatinine				
Other hydration?				

Outcome Assessment

Number or % in study below cut off values defined as malnutrition	Outcome measured*	Outcome number or percentage		Confidence Intervals, Odds ratio, Relative risk, p-value, etc…
		Malnourished	Non-malnourished	

***cardiovascular morbidity (reinfarction, complications), readmission, medium/long-term disability or functional status**

Supplementary Table S3:Critical appraisal of included studies according to SIGN cohort checklist

	Akin 2015	Aronson 2010	Bucholz 2012	Bucholz 2016a	Bucholz 2016b	Colombo 2015	Fukouka 2019	Hoit 1987	Ikeda 2011
The study addresses an appropriate and clearly focused question	Y	Y	Y	Y	Y	Y	Y	Y	Y
The two groups being studied are selected from source populations that are comparable in all respects other than the factor under investigation	Y	Y	Y	Y	Y	Y	Y	Y	Y
The study indicates how many of the people asked to take part did so, in each of the groups being studied	Y	Y	Y	Y	Y	Y	Y	Y	Y
The likelihood that some eligible subjects might have the outcome at the time of enrolment is assessed and taken into account in the analysis.	NA	NA	NA	NA	NA	NA	NA	NA	NA
What percentage of individuals or clusters recruited into each arm of the study dropped out before the study was completed?	E: 0% C: 0%	E: 0% C: 0%	E: 0% C: 0%	E: 0% C: 0%	E: 0% C: 0%	E: 0% C: 0%	C*	E: 0% C: 0%	E: 0% C: 0%
Comparison is made between full participants and those lost to follow up, by exposure status	NA	NA	NA	NA	NA	NA	N	NA	NA
The outcomes are clearly defined	Y	Y	Y	Y	Y	Y	Y	Y	Y
The assessment of outcome is made blind to exposure status. If the study is retrospective this may not be applicable.	N	N	N	N	N	N	N	N	N
Where blinding was not possible, there is some recognition that knowledge of exposure status could have influenced the assessment of outcome	Y	Y	Y	Y	Y	Y	Y	N	C
The method of assessment of exposure is reliable.	N	Y	Y	Y	Y	Y	Y	Y	C

Evidence from other sources is used to demonstrate that the method of outcome assessment is valid and reliable	NA	NA	NA	NA	NA	NA	NA	NA	NA
Exposure level or prognostic factor is assessed more than once.	N	N	N	N	N	N	N	N	N
The main potential confounders are identified and taken into account in the design and analysis	C	Y	Y	Y	Y	Y	Y	N	Y
Have confidence intervals been provided?	N	N	Y	Y	Y	Y	Y	N	Y
How well was the study done to minimise the risk of bias or confounding	0	+	++	++	++	++	+	0	+
Taking into account clinical considerations, your evaluation of the methodology used, and the statistical power of the study, do you think there is clear evidence of an association between exposure and outcome?	N	Y	Y	Y	Y	Y	Y	Y	Y
Are the results of this study directly applicable to the patient group targeted in this guideline?	Y	Y	Y	Y	Y	Y	Y	Y	Y

Summary of SIGN Bias Analysis (Adapted from SIGN guidelines) Y=Yes; N=No; C=Cannot say; NA=Not Applicable; ++ = High Quality; + = Acceptable; 0= low quality; E=Malnourished population as indicated by respective nutrition marker; C=Well-nourished population as indicated by respective nutrition markers

Supplementary Table S4:Critical appraisal of included studies according to SIGN cohort checklist

	Jelavic 2016	Kang 2010	Kim 2019	Kragelund 2005	Li 2013	Lopez-Jimenez 2008	Mehta 2007	Neeland 2017	Nigam 2006
The study addresses an appropriate and clearly focused question	Y	Y	Y	Y	Y	Y	Y	Y	Y
The two groups being studied are selected from source populations that are comparable in all respects other than the factor under investigation	Y	Y	Y	Y	Y	Y	Y	Y	Y
The study indicates how many of the people asked to take part did so, in each of the groups being studied	Y	Y	Y	Y	Y	Y	Y	Y	Y
The likelihood that some eligible subjects might have the outcome at the time of enrolment is assessed and taken into account in the analysis.	NA	NA	NA	NA	NA	Y	NA	Y	NA
What percentage of individuals or clusters recruited into each arm of the study dropped out before the study was completed?	E: 0% C: 0%	Overall 53%	E: 0% C: 0%	Overall: 0.6%	Overall 2.4%	E: 0% C: 0%	E: 0% C: 0%	E: 0% C: 0%	E: 0% C: 0%
Comparison is made between full participants and those lost to follow up, by exposure status	NA	C	NA	NA	C	NA	NA	NA	NA
The outcomes are clearly defined	Y	Y	Y	Y	Y	Y	Y	Y	Y
The assessment of outcome is made blind to exposure status. If the study is retrospective this may not be applicable.	N	N	N	N	N	N	N	N	N

Where blinding was not possible, there is some recognition that knowledge of exposure status could have influenced the assessment of outcome	C	N	Y	Y	N	Y	Y	Y	Y
The method of assessment of exposure is reliable.	Y	Y	Y	Y	Y	Y	N	Y	Y
Evidence from other sources is used to demonstrate that the method of outcome assessment is valid and reliable	NA	NA	NA	NA	NA	NA	NA	Y	NA
Exposure level or prognostic factor is assessed more than once.	N	N	N	N	Y	Y	N	N	N
The main potential confounders are identified and taken into account in the design and analysis	C	Y	Y	Y	N	Y	Y	Y	Y
Have confidence intervals been provided?	N	Y	Y	Y	N	Y	N	Y	Y
How well was the study done to minimise the risk of bias or confounding	0	+	++	++	+	++	+	++	++
Taking into account clinical considerations, your evaluation of the methodology used, and the statistical power of the study, do you think there is clear evidence of an association between exposure and outcome?	N	Y	Y	Y	Y	Y	N	Y	Y
Are the results of this study directly applicable to the patient group targeted in this guideline?	Y	Y	Y	Y	Y	Y	Y	Y	Y

Summary of SIGN Bias Analysis (Adapted from SIGN guidelines) Y=Yes; N=No; C=Cannot say; NA=Not Applicable; ++ = High Quality; + = Acceptable; 0= low quality; E=Malnourished population as indicated by respective nutrition marker; C=Well-nourished population as indicated by respective nutrition markers

Supplementary Table S5:Critical appraisal of included studies according to SIGN cohort checklist

	Nikolsky 2006	O'Brien 2013	Rana 2004	Rea 2001	Samanta 2020	Wienbergen 2008	Wu 2010	Yokoyama 2019	Zeller 2008
The study addresses an appropriate and clearly focused question	Y	Y	Y	Y	Y	Y	Y	Y	Y
The two groups being studied are selected from source populations that are comparable in all respects other than the factor under investigation	Y	Y	Y	Y	Y	Y	Y	Y	Y
The study indicates how many of the people asked to take part did so, in each of the groups being studied	Y	Y	Y	Y	Y	Y	Y	Y	Y
The likelihood that some eligible subjects might have the outcome at the time of enrolment is assessed and taken into account in the analysis.	NA	NA	NA	NA	NA	NA	NA	NA	NA
What percentage of individuals or clusters recruited into each arm of the study dropped out before the study was completed?	E: 0% C: 0%	E: 0% C: 0%	E: 0% C: 0%	C	E: 0% C: 0%	Overall 25%	E: 0% C: 0%	E: 0% C: 0%	E: 0% C: 0%
Comparison is made between full participants and those lost to follow up, by exposure status	NA	NA	NA	NA	NA	C	NA	NA	NA
The outcomes are clearly defined	Y	Y	Y	Y	Y	Y	Y	Y	Y
The assessment of outcome is made blind to exposure status. If the study is retrospective this may not be applicable.	Y	N	Y	N	Y	N	Y	Y	N
Where blinding was not possible, there is some recognition that knowledge of exposure status could have influenced the assessment of outcome	NA	Y	NA	Y	NA	N	NA	NA	Y
The method of assessment of exposure is reliable.	Y	Y	Y	Y	Y	Y	Y	Y	Y

Evidence from other sources is used to demonstrate that the method of outcome assessment is valid and reliable	NA	NA	NA	NA	NA	NA	NA	NA	NA
Exposure level or prognostic factor is assessed more than once.	N	N	N	N	N	N	N	N	N
The main potential confounders are identified and taken into account in the design and analysis	Y	Y	Y	Y	Y	Y	Y	Y	Y
Have confidence intervals been provided?	Y	Y	Y	N	Y	Y	Y	Y	Y
How well was the study done to minimise the risk of bias or confounding	++	++	++	+	++	++	++	++	++
Taking into account clinical considerations, your evaluation of the methodology used, and the statistical power of the study, do you think there is clear evidence of an association between exposure and outcome?	Y	Y	Y	Y	Y	Y	Y	Y	N
Are the results of this study directly applicable to the patient group targeted in this guideline?	Y	Y	Y	Y	Y	Y	Y	Y	Y

Summary of SIGN Bias Analysis (Adapted from SIGN guidelines) Y=Yes; N=No; C=Cannot say; NA=Not Applicable; ++ = High Quality; + = Acceptable; 0= low quality; E=Malnourished population as indicated by respective nutrition marker; C=Well-nourished population as indicated by respective nutrition markers

Supplementary Table S6: Impact of BMI on mortality in patients following myocardial infarction with corresponding unadjusted and adjusted risk estimates

Study	Effect	Unadjusted (95% CI)	p-value	Adjusted (95%CI)	Adjusted *p*-value	Extreme Group (n)	Comparison Group (n)
Overweight and Mortality						**>25 kg/m^2**	**18.5-24.9 kg/m^2**
Akin 2015	OR	0.91 (0.53-1.55)	-	-	-	432	263
Aronson 2010	OR	0.65 (0.50-0.84)	-	-	-	953	618
Bucholz 2012	HR	0.68 (0.54–0.86)	-	0.76 (0.59–0.97)	-	2314	1449
Bucholz 2016a	HR	0.76 (0.75-0.77)	-	0.91 (0.89-0.92)	-	48422	51896
Colombo 2015 (Diabetes)	HR	0.76 (0.56-1.02)	0.0707	0.83 (0.61-1.13)	0.2383	516	226
Colombo 2015 (No Diabetes)	HR	0.74 (0.59-0.93)	0.0097	0.73 (0.58-0.93)	0.0087	1411	848
Fukuoka 2019 (over 70 years old)	HR	-	-	0.72 (0.38-1.39)	0.33	145	476
Fukuoka 2019 (under 70 years old)	HR	-	-	3.82 (1.49-9.79)	0.005	347	443
Hoit 1987	OR	0.82 (0.60-1.12)	-	-	-	884	658
Ikeda 2011	OR	0.55 (0.17-1.86)	-	-	-	46	75

Jelavić 2016	OR	0.16 (0.02-1.60)	-	-	-	118	60
Kim 2019	OR	0.60 (0.47-0.78)	-	-	-	2568	5368
Kragelund 2004 (men)	OR	0.80 (0.70-0.91)	-	-	-	1996	1613
Kragelund 2004 (women)	OR	0.74 (0.61-0.91)	-	-	-	610	989
Li 2013	OR	1.16 (0.84-1.60)	-	-	-	718	476
Lopez-Jimenez 2008	HR	-	-	0.96 (0.69-1.34)	0.8	872	528
Mehta 2007	OR	0.69 (0.48-0.99)	-	-	-	1093	703
Neeland 2017	OR	0.86 (0.80-0.92)	-	-	-	7982	5920
Nigam 2006	HR	0.74 (0.55, 0.99)	< 0.01	0.71 (0.51-0.97)	<0.01	366	236
Nikolsky 2006	OR	0.48 (0.30-0.77)	-	-	-	915	552
O’Brien 2014	HR	0.62 (0.60-0.65)	-	0.78 (0.75-0.81)	-	12506	11186
Rana 2004	HR	0.54 (0.50-0.59)	<0.05	1.14 (0.80-1.62)	<0.05	832	607
Samanta 2020	OR	0.39 (0.18-0.86)	-	-	-	183	164
Wienbergen 2007	OR	0.64 (0.53-0.77)	-	-	-	3744	2457
Yokoyama 2019	OR	0.34 (0.15-0.77)	-	-	-	134	126

Obesity and Mortality						**>30 kg/m²**	**18.5-24.9 kg/m²**
Akin 2015	OR	0.65 (0.32-1.34)	-	-	-	195	263
Aronson 2010 (obese*)	OR	0.68 (0.50-0.94)	-	-	-	434	618
Aronson 2010 (morbidly obese*)	OR	1.31 (0.84-2.03)	-	-	-	126	618
Bucholz 2012 (obese*)	HR	0.54 (0.40–0.71)	-	0.62 (0.45–0.84)	-	1531	1449
Bucholz 2012 (morbidly obese*)	HR	0.49 (0.35–0.69)	-	0.56 (0.39–0.81)	-	1065	1449
Bucholz 2016 (obese*)	HR	0.77 (0.75-0.78)	-	0.93 (0.92-0.95)	-	17822	51896
Bucholz 2016 (morbidly obese*)	HR	0.83 (0.81-0.86)	-	0.98 (0.96-1.01)	-	6841	51896
Colombo 2015 (Diabetes*)*	HR	0.79 (0.58-1.09)	0.1495	0.98 (0.71-1.36)	0.8914	448	226
Colombo 2015 (No Diabetes*)*	HR	0.62 (0.46-0.85)	0.0023	0.64 (0.47-0.87)	0.0043	605	848
Hoit 1987	OR	0.49 (0.28, 0.87)	-	-	-	218	658
Jelavić 2016	OR	0.13 (0.01-2.69)	-	-	-	72	60
Kragelund 2004 (men)	OR	0.85 (0.70-1.03)	-	-	-	544	1613
Kragelund 2004 (women)	OR	0.69 (0.52-0.91)	-	-	-	255	989
Li 2013	OR	0.94 (0.58-1.53)	-	-	-	186	476

Lopez-Jimenez 2008	HR	-	-	0.74 (0.51-1.08)	0.1	700	528
Mehta 2007	OR	0.44 (0.26-0.72)	-	-	-	583	703
Neeland 2017	OR	0.89 (0.83-0.96)	-	-	-	3837	5920
Nigam 2006	HR	0.62 (0.51-1.10)	0.02	0.61 (0.42-0.89)	0.02	292	236
Nikolsky 2006	OR	0.22 (0.11-0.44)	-	-	-	569	552
O'Brien 2014 (Class I**)	HR	0.53 (0.51-0.56)	-	0.75 (0.70-0.79)	-	6089	11186
O'Brien 2014 (Class II**)	HR	0.57 (0.52-0.61)	-	0.83 (0.76-0.90)	-	2226	11186
O'Brien 2014 (Class III**)	HR	0.65 (0.58-0.71)	-	0.93 (0.83-1.03)	-	1222	11186
Rana 2004 (Class I**)	HR	NA	<0.05	1.44 (0.94-2.22)	0.08	331	607
Rana 2004 (Class II and III**)	HR	NA	<0.05	1.50 (0.80-2.80)	0.08	128	607
Samanta 2020	OR	0.27 (0.10-0.75)	-	-	-	129	164
Wienbergen 2007	OR	0.50 (0.39-0.66)	-	0.56 (0.40-0.79)	-	1635	2457
Yokoyama 2019	OR	0.22 (0.09-0.57)	-	-	-	134	126
Underweight and Mortality						**<18.5 kg/m²**	**18.5-24.9 kg/m²**
Aronson 2010	OR	4.30 (1.94-9.51)	-	-	-	26	618

Bucholz 2016b	HR	1.67 (1.62, 1.71)	-	1.26 (1.23, 1.30)	-	5678	51896
Fukuoka 2019 (over 70 years old)	HR	-	-	1.69 (1.12-2.55)	0.012	162	476
Fukuoka 2019 (under 70 years old)	HR	-	-	0.43 (0.05-3.73)	0.44	61	443
Kim 2019	HR	3.42 (2.65-4.41)	<0.001	1.54 (1.18-2.01)	0.002	2632	5368
Kragelund 2004 (men)	OR	2.50 (1.22-5.13)	-	-	-	41	1613
Kragelund 2004 (women)	OR	2.55 (1.58-4.14)	-	-	-	120	989
Lopez-Jimenez 2008	HR	NA	-	1.77 (1.00-3.12)	0.05	84	528
O'Brien 2014	HR	1.66 (1.53-1.79)	-	1.48 (1.38-1.60)	-	1236	11186
Yokoyama 2019	OR	1.66 (0.90-3.06)	-	-	-	123	126

** Obese refers to BMI of 30–34.9kg/m^2 and morbidly obese refers to BMI of ≥35kg/m^2*
***Class 1 refers to BMI of 30–34.9kg/m^2, Class 2 refers to BMI of 35–39.9kg/m^2 and Class 3 refers to BMI of >40kg/m^2*

Supplementary Table S7: Impact of BMI on recurrence in patients following myocardial infarction with corresponding unadjusted and adjusted risk estimates

Study	Effect	Unadjusted (95% CI)	p-value	Adjusted (95%CI)	Adjusted p-value	Extreme Group (n)	Comparison Group (n)
Overweight and Recurrent Event						**>25 kg/m^2**	**18.5-24.9 kg/m^2**
Akin 2015	OR	0.80 (0.40-1.60)	-	-	-	432	263
Ikeda 2011	OR	2.55 (0.41-15.85)	-	-	-	46	75
Hoit 1987	OR	0.98 (0.68-1.42)	-	-	-	884	658
Jelavić 2016	OR	0.25 (0.01-7.56)	-	-	-	118	60
Kim 2019	OR	0.81 (0.54-1.23)	-	-	-	2568	5368
Li 2013	OR	0.87 (0.45-1.68)	-	-	-	718	476
Lopez-Jimenez 2008	OR	0.99 (0.72-1.35)	-	-	-	872	528
Mehta 2007	OR	0.73 (0.48-1.10)	-	-	-	1039	703
Nigam 2006	HR	2.28 (1.20-4.35)	0.03	2.59 (1.31-5.12)	0.01	366	236
Rea 2001	OR	0.84 (0.65-1.08)	-	-	-	460	798
Samanta 2020	OR	1.16 (0.42-3.19)	-	-	-	183	164
Obesity and Recurrent Event						**>30 kg/m^2**	**18.5-24.9 kg/m^2**
Akin 2015	OR	0.89 (0.39-2.03)	-	-	-	195	263

Hoit 1987	OR	0.84 (0.47-1.53)	-	-	-	218	658
Jelavić 2016	OR	0.83 (0.05-13.57)	-	-	-	72	60
Li 2013	OR	1.63 (0.73-3.67)	-	-	-	186	476
Lopez-Jimenez 2008	OR	0.99 (0.72-1.35)	-	-	-	834	528
Mehta 2007	OR	0.73 (0.44-1.19)	-	-	-	583	703
Nigam 2006	HR	1.56 (0.77, 3.16)	0.02	1.83 (0.86-3.90)	0.02	292	236
Rea 2001 (Class I*)	OR	1.08 (0.80-1.47)	-	-	-	461	798
Rea 2001 (Class II and Class III*)	OR	1.63 (1.15-2.31)	-	-	-	245	798
Samanta 2020	OR	1.48 (0.52-4.20)	-	-	-	129	164
Underweight and Recurrent Event						**<18.5 kg/m²**	**18.5-24.9 kg/m²**
Kim 2019	OR	1.22 (0.86-1.75)	-	-	-	2632	5368
Lopez-Jimenez 2008	OR	0.94 (0.48-1.85)	-	-	-	84	528

***Class 1 refers to BMI of 30-34.9kg/m², Class 2 refers to BMI of 35-39.9kg/m² and Class 3 refers to BMI of >40kg/m²*

Supplementary Table S8: Impact of BMI on hospital readmission in patients following myocardial infarction with corresponding unadjusted and adjusted risk estimate

**Study**	**Effect**	**Unadjusted (95% CI)**	**p-value**	**Adjusted (95%CI)**	**Adjusted p-value**	**Extreme Group (n)**	**Comparison Group (n)**
Overweight and Readmission						**>25 kg/m^2**	**18.5-24.9 kg/m^2**
**Ikeda 2011**	OR	0.31 (0.14-0.68)	NA	NA	NA	46	75
**Jelavić 2016**	OR	0.78 (0.36-1.70)	NA	NA	NA	118	60
**Li 2013**	OR	1.32 (0.93, 1.88)	-	-	-	718	476
**O'Brien 2013**	HR	0.85 (0.83-0.88)	NA	0.94 (0.91-0.97)	NA	12506	11186
Obesity and Readmission						**>30 kg/m^2**	**18.5-24.9 kg/m^2**
**Jelavić 2016**	OR	0.87 (0.37-2.04)	NA	NA	NA	72	60
**Li 2013**	OR	0.75 (0.42, 1.35)	-	-	-	186	476
**O'Brien 2013 (Class I*)**	HR	0.85 (0.82-0.89)	NA	0.97 (0.94-1.01)	NA	6089	11186
**O'Brien 2013 (Class II*)**	HR	0.94 (0.89-0.99)	NA	1.06 (1.01-1.12)	NA	2226	11186
**O'Brien 2013**	HR	0.98 (0.92-1.05)	NA	1.05 (0.98-1.13)	NA	1222	11186

**(Class III*)**							
Underweight and Readmission						**<18.5 kg/m^2**	**18.5-24.9 kg/m^2**
**O'Brien 2013**	HR	1.16 (1.08-1.25)	NA	1.08 (1.00-1.16)	NA	1236	11186

Supplementary Figure S1: Formulas for unadjusted odds ratio and 95% confidence intervals (adapted from Heidel, 2021)

	Event	*No Event*
Exposure	**A**	**B**
Control	**C**	**D**

$$Odds\ Ratio = \frac{(A \times D}{(B \times C)}$$

$$95\%\ CI = OR \pm 1.96 \times \sqrt{\frac{1}{A} + \frac{1}{B} + \frac{1}{C} + \frac{1}{D}}$$

Supplementary Figure S2: Funnel Plot for the primary outcome of mortality following MI in overweight, obese, morbidly obese and underweight patients.

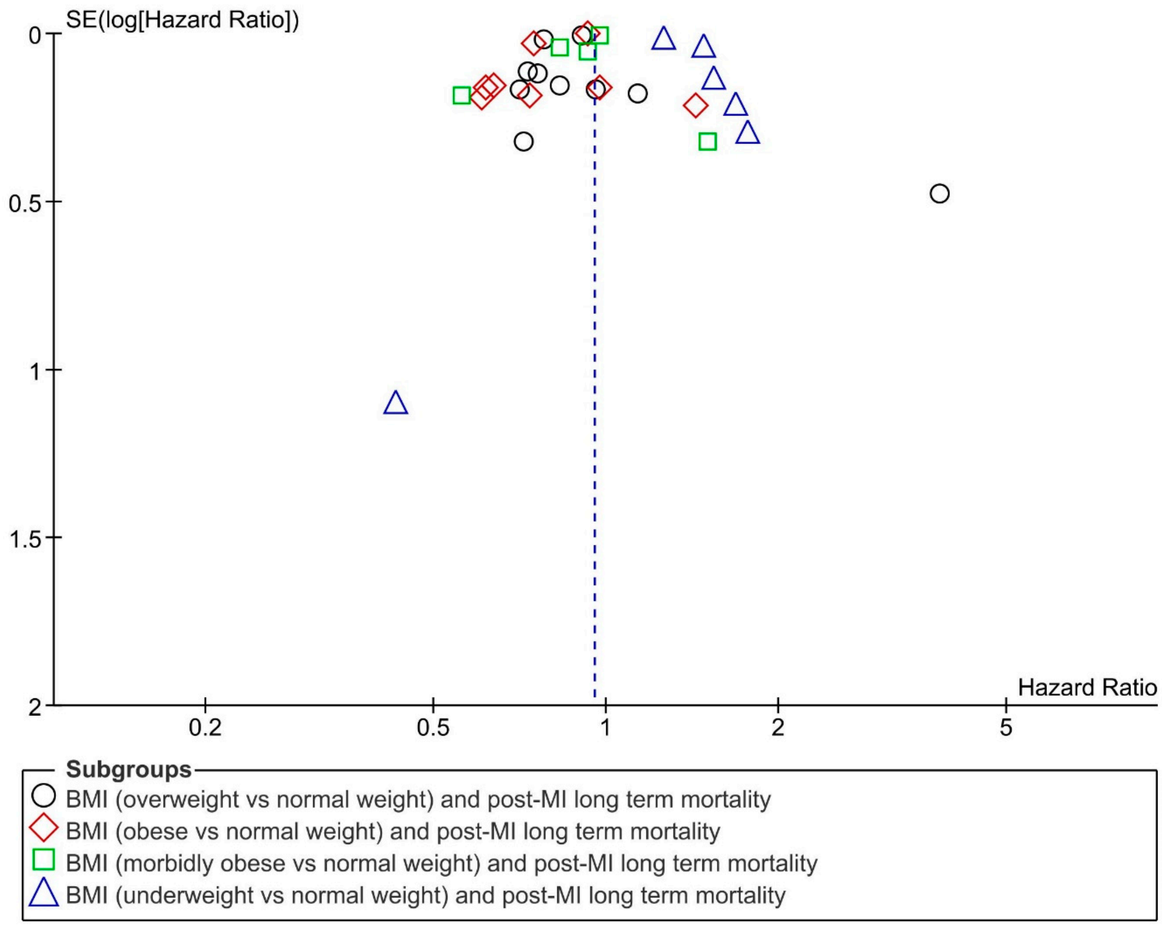

Symmetry of the funnel plot is visible suggesting a low risk of publication bias.

Supplementary Figure S3: Forest plot displaying the risk of long-term mortality following MI in overweight (BMI 25–29.9 kg/m^2) patients compared to normal weight (BMI 18.5–24.9 kg/m^2) for the studies using unadjusted hazard ratios.

BMI (overweight vs normal weight) and post-MI long-term mortality – unadjusted HRs

Study or Subgroup	log[Hazard Ratio]	SE	Overweight Total	Normal weight Total	Weight	Hazard Ratio IV, Random, 95% CI	Year
Rana 2004	-0.6162	0.0393	832	607	18.0%	0.54 [0.50, 0.58]	2004
Nigam 2006	-0.3011	0.1514	366	236	9.8%	0.74 [0.55, 1.00]	2006
Bucholz 2012	-0.3857	0.1176	2314	1449	12.2%	0.68 [0.54, 0.86]	2012
O'Brien 2014	-0.478	0.0167	12506	11186	19.0%	0.62 [0.60, 0.64]	2013
Colombo 2015 (No diabetes)	-0.3011	0.1156	1411	848	12.3%	0.74 [0.59, 0.93]	2015
Colombo 2015 (Diabetes)	-0.2744	0.1558	516	226	9.5%	0.76 [0.56, 1.03]	2015
Bucholz 2016a	-0.2744	0.0068	48422	51896	19.2%	0.76 [0.75, 0.77]	2016
**Total (95% CI)**			**66367**	**66448**	**100.0%**	**0.67 [0.59, 0.77]**	

Heterogeneity: Tau² = 0.02; Chi² = 189.75, df = 6 (P < 0.00001); I² = 97%
Test for overall effect: Z = 5.81 (P < 0.00001)

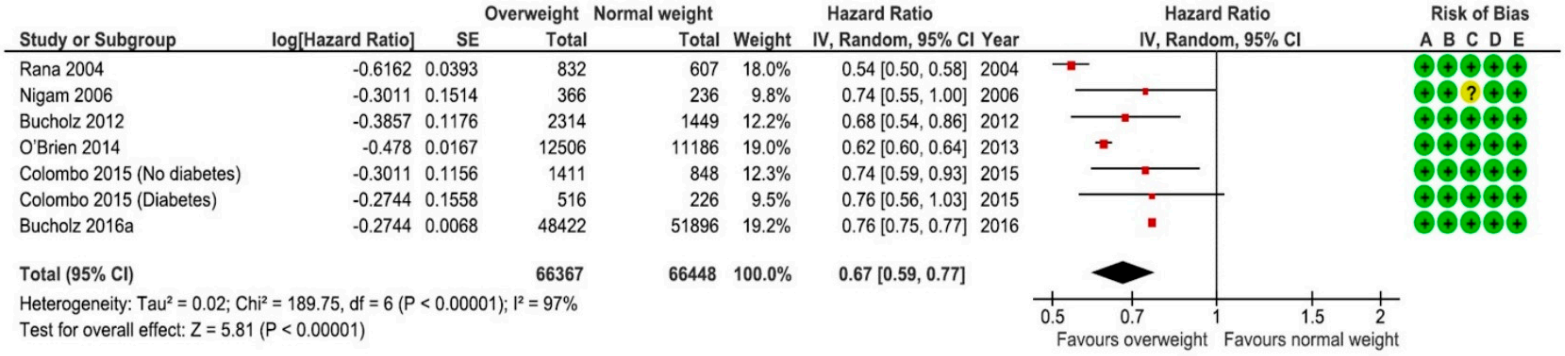

Risk of bias legend
(**A**) Selection Bias
(**B**) Performance Bias
(**C**) Attrition Bias
(**D**) Detection Bias
(**E**) Overall study quality (+ = High quality; ? = Acceptable; - = Low quality)

The risk of bias summary produced from the critical appraisal according to the SIGN cohort appraisal checklist is also displayed for each included study.

Supplementary Figure S4 : Forest plot displaying the risk of long-term mortality following MI in overweight (BMI 25–29.9 kg/m^2) patients compared to normal weight (BMI 18.5–24.9 kg/m^2) for the studies using unadjusted odds ratio.

BMI (overweight vs normal weight) and post-MI long-term mortality – unadjusted ORs

Study or Subgroup	log[Odds Ratio]	SE	Overweight Total	Normal weight Total	Weight	Odds Ratio IV, Random, 95% CI	Risk of Bias A	B	C	D	E
Akin 2015	-0.0943	0.2855	432	263	3.4%	0.91 [0.52, 1.59]	?	+	+	-	-
Aronson 2010	-0.4308	0.1336	953	618	8.7%	0.65 [0.50, 0.84]	+	+	+	+	+
Hoit 1987	-0.1987	0.158	884	658	7.4%	0.82 [0.60, 1.12]	+	+	+	-	-
Ikeda 2011	-0.5978	0.5991	46	75	0.9%	0.55 [0.17, 1.78]	+	+	+	-	?
Jelavić 2016	-1.8326	1.061	118	60	0.3%	0.16 [0.02, 1.28]	+	+	+	-	-
Kim 2019	-0.5028	0.132	2568	5368	8.8%	0.60 [0.47, 0.78]	+	+	+	+	+
Kragelund 2005 (men)	-0.2231	0.0681	1996	1613	12.7%	0.80 [0.70, 0.91]	+	+	+	+	+
Kragelund 2005 (women)	-0.3011	0.0986	610	989	10.8%	0.74 [0.61, 0.90]	+	+	+	+	+
Li 2013	0.1518	0.1636	718	476	7.1%	1.16 [0.84, 1.60]	+	+	+	?	?
Mehta 2007	-0.3711	0.1852	1030	703	6.2%	0.69 [0.48, 0.99]	+	+	+	-	?
Neeland 2017	-0.1511	0.035	7982	5920	14.5%	0.86 [0.80, 0.92]	+	+	+	+	+
Nikolsky 2006	-0.734	0.2398	915	552	4.4%	0.48 [0.30, 0.77]	+	+	+	+	+
Samanta 2020	-0.9324	0.4005	183	164	2.0%	0.39 [0.18, 0.86]	+	+	+	+	+
Wienbergen 2008	-0.4506	0.098	3744	2457	10.8%	0.64 [0.53, 0.77]	+	+	-	+	?
Yokoyama 2019	-1.0777	0.4174	134	126	1.8%	0.34 [0.15, 0.77]	+	+	+	+	+
**Total (95% CI)**			**22313**	**20042**	**100.0%**	**0.72 [0.64, 0.81]**					

Heterogeneity: Tau² = 0.02; Chi² = 37.78, df = 14 (P = 0.0006); I² = 63%
Test for overall effect: Z = 5.42 (P < 0.00001)

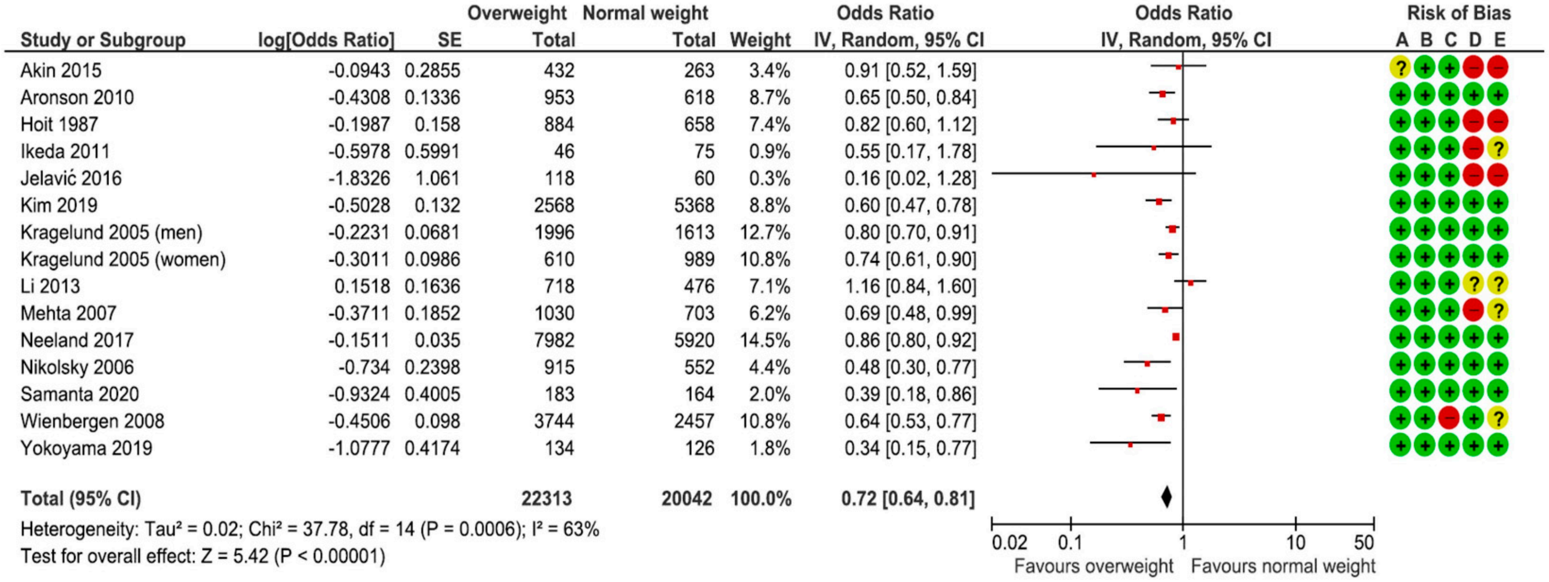

Risk of bias legend
(**A**) Selection Bias
(**B**) Performance Bias
(**C**) Attrition Bias
(**D**) Detection Bias
(**E**) Overall study quality (+ = High quality; ? = Acceptable; - = Low quality)

The risk of bias summary produced from the critical appraisal according to the SIGN cohort appraisal checklist is also displayed for each included study.

Supplementary Figure S5: Forest plots displaying the risk of long-term mortality following MI in overweight (BMI 25–29.9 kg/m^2), obese (BMI ≥ 30 kg/m^2), morbidly obese (BMI ≥35 kg/m^2) and underweight (BMI < 18.5 kg/m^2) patients compared to normal weight (BMI 18.5–24.9 kg/m^2) for the studies using adjusted hazard ratio following sensitivity analysis.

BMI and post-MI long term mortality – adjusted HRs following sensitivity analysis

Study or Subgroup	log[Hazard Ratio]	SE	Expsoure Total	Comparison Total	Weight	Hazard Ratio IV, Random, 95% CI	Risk of Bias A	B	C	D	E
**4.1.1 BMI (overweight vs normal weight) and post-MI long term mortality**											
Bucholz 2012	-0.2744	0.1245	2314	1449	21.9%	0.76 [0.60, 0.97]	+	+	+	+	+
Colombo 2015 (Diabetes)	-0.1863	0.1571	516	226	14.3%	0.83 [0.61, 1.13]	+	+	+	+	+
Colombo 2015 (No diabetes)	-0.3147	0.1174	1411	848	24.3%	0.73 [0.58, 0.92]	+	+	+	+	+
Fukuoka 2019 (over 70 years old)	-0.3285	0.3261	145	476	3.5%	0.72 [0.38, 1.36]	+	+	-	+	?
Lopez-Jimenez 2008	-0.0408	0.1685	872	528	12.5%	0.96 [0.69, 1.34]	+	+	+	+	+
Nigam 2006	-0.3425	0.1688	366	236	12.5%	0.71 [0.51, 0.99]	+	+	?	+	+
Rana 2004	0.131	0.1807	832	607	11.0%	1.14 [0.80, 1.62]	+	+	+	+	+
**Subtotal (95% CI)**			**6456**	**4370**	**100.0%**	**0.81 [0.72, 0.92]**					
Heterogeneity: Tau² = 0.00; Chi² = 6.40, df = 6 (P = 0.38); I² = 6%											
Test for overall effect: Z = 3.39 (P = 0.0007)											
**4.1.2 BMI (obese vs normal weight) and post-MI long term mortality**											
Bucholz 2012 (morbidly obese)	-0.5798	0.1846	1065	1449	13.0%	0.56 [0.39, 0.80]	+	+	+	+	+
Bucholz 2012 (obese)	-0.478	0.1635	1531	1449	13.9%	0.62 [0.45, 0.85]	+	+	+	+	+
Colombo 2015 (Diabetes)	-0.0202	0.1644	448	226	13.9%	0.98 [0.71, 1.35]	+	+	+	+	+
Colombo 2015 (No diabetes)	-0.4463	0.1575	605	848	14.2%	0.64 [0.47, 0.87]	+	+	+	+	+
Lopez-Jimenez 2008	-0.3011	0.1899	700	528	12.8%	0.74 [0.51, 1.07]	+	+	+	+	+
Nigam 2006	-0.4943	0.1904	292	236	12.7%	0.61 [0.42, 0.89]	+	+	?	+	+
Rana 2004 (Class I)	0.3646	0.2176	331	607	11.6%	1.44 [0.94, 2.21]	+	+	+	+	+
Rana 2004 (Class II and III)	0.4055	0.3207	128	607	8.0%	1.50 [0.80, 2.81]	+	+	+	+	+
**Subtotal (95% CI)**			**5100**	**5950**	**100.0%**	**0.79 [0.63, 1.00]**					
Heterogeneity: Tau² = 0.08; Chi² = 22.34, df = 7 (P = 0.002); I² = 69%											
Test for overall effect: Z = 1.95 (P = 0.05)											
**4.1.3 BMI (underweight vs normal weight) and post-MI long term mortality**											
Fukuoka 2019 (over 70 years old)	0.5247	0.2099	162	476	25.6%	1.69 [1.12, 2.55]	+	+	-	+	?
Kim 2019	0.4318	0.1359	2632	5368	61.1%	1.54 [1.18, 2.01]	+	+	+	+	+
Lopez-Jimenez 2008	0.571	0.2913	84	528	13.3%	1.77 [1.00, 3.13]	+	+	+	+	+
**Subtotal (95% CI)**			**2878**	**6372**	**100.0%**	**1.61 [1.30, 1.98]**					
Heterogeneity: Tau² = 0.00; Chi² = 0.27, df = 2 (P = 0.88); I² = 0%											
Test for overall effect: Z = 4.46 (P < 0.00001)											

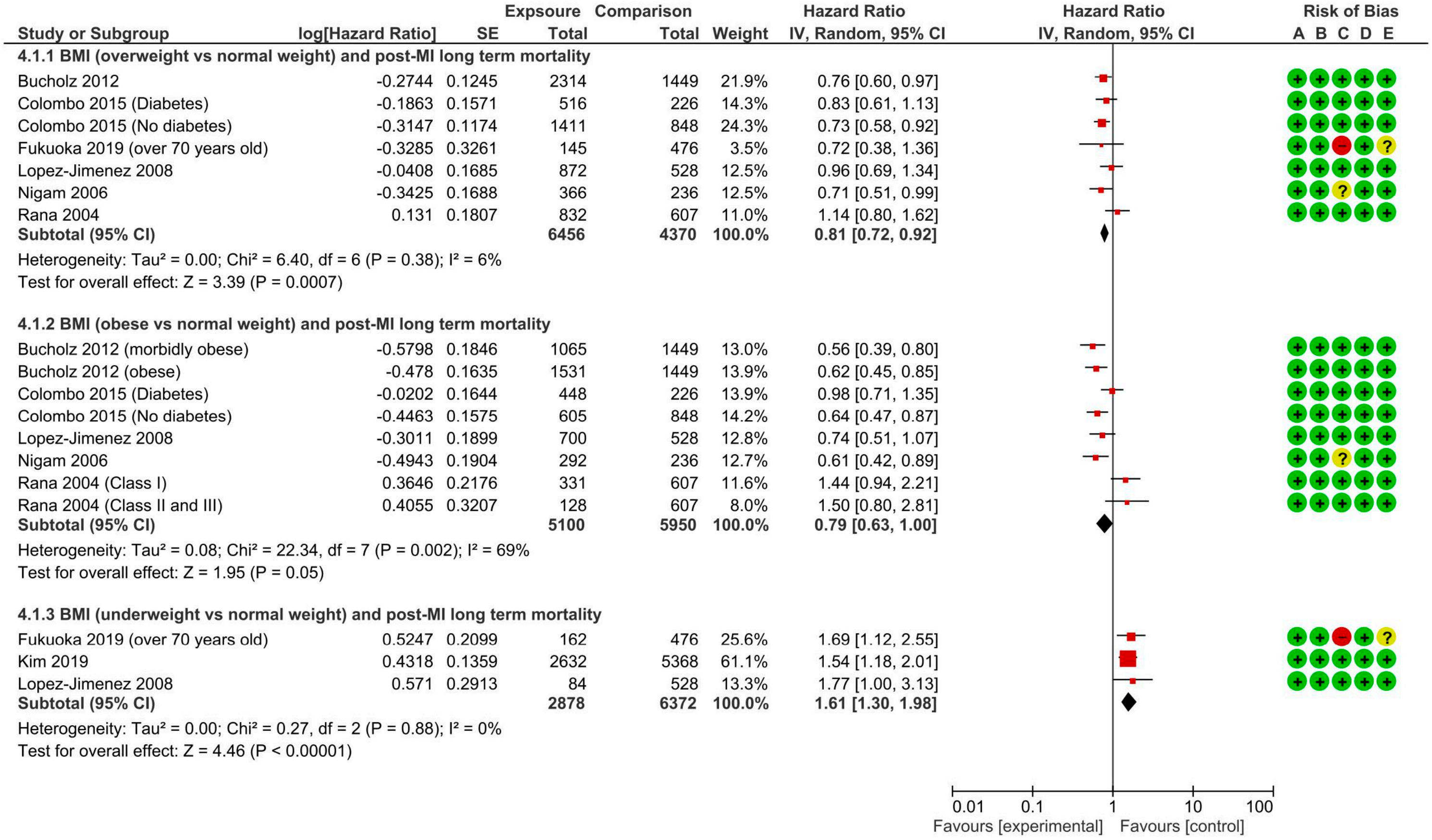

Risk of bias legend
(**A**) Selection Bias
(**B**) Performance Bias
(**C**) Attrition Bias
(**D**) Detection Bias
(**E**) Overall study quality (+ = High quality; ? = Acceptable; - = Low quality)

The risk of bias summary produced from the critical appraisal according to the SIGN cohort appraisal checklist is also displayed for each included study.

Supplementary Figure S6: Forest plot displaying the risk of long-term mortality following MI in obese (BMI ≥ 30 kg/m^2) patients compared to normal weight (BMI 18.5–24.9 kg/m^2) for the studies using unadjusted hazard ratio.

BMI (obese vs normal weight) and post-MI long-term mortality – unadjusted HRs

Study or Subgroup	log[Hazard Ratio]	SE	Obese Total	Normal weight Total	Weight	Hazard Ratio IV, Random, 95% CI	Risk of Bias A	B	C	D	E
Bucholz 2012 (morbidly obese)	-0.7133	0.1717	1065	1449	7.1%	0.49 [0.35, 0.69]	+	+	+	+	+
Bucholz 2012 (obese)	-0.6162	0.1531	1531	1449	7.8%	0.54 [0.40, 0.73]	+	+	+	+	+
Bucholz 2016 (obese)	-0.1863	0.0124	6841	51896	12.3%	0.83 [0.81, 0.85]	+	+	+	+	+
Bucholz 2016a (morbidly obese)	-0.2614	0.0134	17822	51896	12.3%	0.77 [0.75, 0.79]	+	+	+	+	+
Colombo 2015 (Diabetes)	-0.2357	0.1577	448	226	7.6%	0.79 [0.58, 1.08]	+	+	+	+	+
Colombo 2015 (No diabetes)	-0.478	0.1523	605	848	7.8%	0.62 [0.46, 0.84]	+	+	+	+	+
Nigam 2006	-0.478	0.0996	293	236	9.9%	0.62 [0.51, 0.75]	+	+	?	+	+
O'Brien 2014 (Class I)	-0.6349	0.0196	6089	11186	12.2%	0.53 [0.51, 0.55]	+	+	+	+	+
O'Brien 2014 (Class II)	-0.5621	0.0468	2226	11186	11.7%	0.57 [0.52, 0.62]	+	+	+	+	+
O'Brien 2014 (Class III)	-0.4308	0.0581	1222	11186	11.4%	0.65 [0.58, 0.73]	+	+	+	+	+
**Total (95% CI)**			**38142**	**141558**	**100.0%**	**0.64 [0.56, 0.73]**					

Heterogeneity: Tau² = 0.04; Chi² = 434.53, df = 9 (P < 0.00001); I² = 98%

Test for overall effect: Z = 6.36 (P < 0.00001)

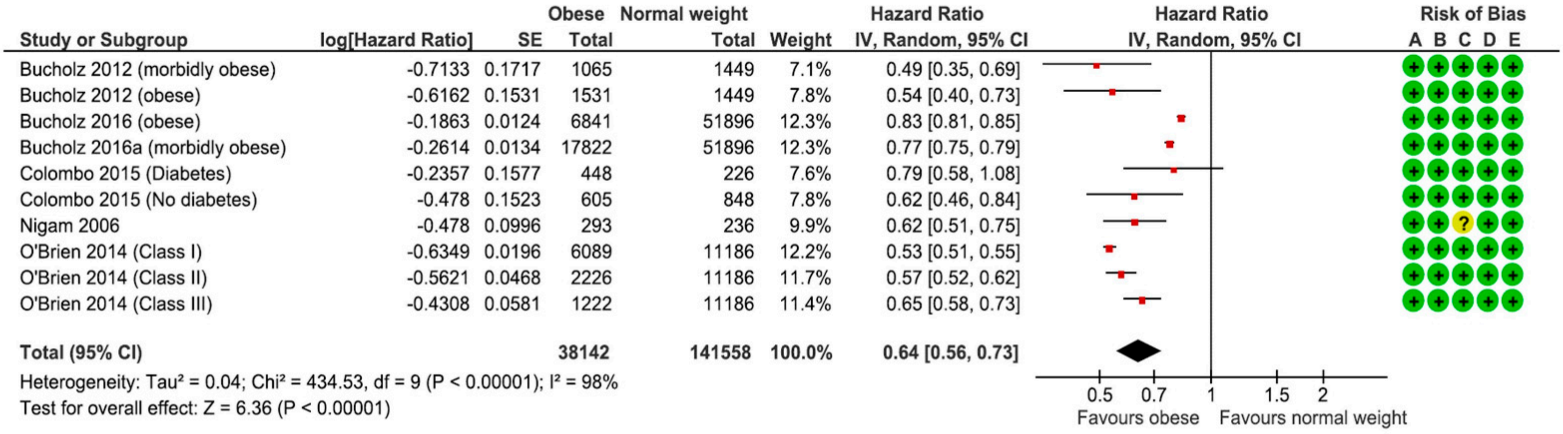

Risk of bias legend

(**A**) Selection Bias
(**B**) Performance Bias
(**C**) Attrition Bias
(**D**) Detection Bias
(**E**) Overall study quality (+ = High quality; ? = Acceptable; - = Low quality)

The risk of bias summary produced from the critical appraisal according to the SIGN cohort appraisal checklist is also displayed for each included study.

Supplementary Figure S7: Forest plot displaying the risk of long-term mortality following MI in obese (BMI ≥ 30 kg/m^2) patients compared to normal weight (BMI 18.5–24.9 kg/m^2) for the studies using unadjusted odds ratio.

BMI (obese vs normal weight) and post-MI long-term mortality – unadjusted ORs

Study or Subgroup	log[Odds Ratio]	SE	Obese Total	Normal weight Total	Weight	Odds Ratio IV, Random, 95% CI	Risk of Bias A	B	C	D	E
Akin 2015	-0.4308	0.3616	195	263	5.1%	0.65 [0.32, 1.32]	?	+	+	-	-
Aronson 2010 (morbidly obese)	0.2673	0.2251	126	618	8.0%	1.31 [0.84, 2.03]	+	+	+	+	+
Aronson 2010 (obese)	-0.3797	0.1645	434	618	9.5%	0.68 [0.50, 0.94]	+	+	+	+	+
Hoit 1987	-0.7104	0.2915	218	658	6.4%	0.49 [0.28, 0.87]	+	+	+	-	-
Jelavić 2016	-2.0402	1.3087	72	60	0.6%	0.13 [0.01, 1.69]	+	+	+	-	-
Kragelund 2005 (men)	-0.1625	0.0991	544	1613	11.1%	0.85 [0.70, 1.03]	+	+	+	+	+
Kragelund 2005 (women)	-0.3711	0.1443	255	989	10.0%	0.69 [0.52, 0.92]	+	+	+	+	+
Li 2013	-0.0592	0.2479	186	476	7.4%	0.94 [0.58, 1.53]	+	+	+	?	?
Mehta 2007	-0.821	0.2513	583	703	7.3%	0.44 [0.27, 0.72]	+	+	+	-	?
Neeland 2017	-0.1127	0.0381	5597	5920	12.1%	0.89 [0.83, 0.96]	+	+	+	+	+
Nikolsky 2006	-1.5141	0.3537	569	522	5.3%	0.22 [0.11, 0.44]	+	+	+	+	+
Samanta 2020	-1.2925	0.5125	129	164	3.2%	0.27 [0.10, 0.75]	+	+	+	+	+
Wienbergen 2008	-0.6859	0.1349	1635	2457	10.3%	0.50 [0.39, 0.66]	+	+	-	+	?
Yokoyama 2019	-1.5069	0.4791	134	126	3.6%	0.22 [0.09, 0.57]	+	+	+	+	+
**Total (95% CI)**			**10677**	**15187**	**100.0%**	**0.62 [0.50, 0.77]**					

Heterogeneity: Tau² = 0.09; Chi² = 62.92, df = 13 (P < 0.00001); I² = 79%
Test for overall effect: Z = 4.43 (P < 0.00001)

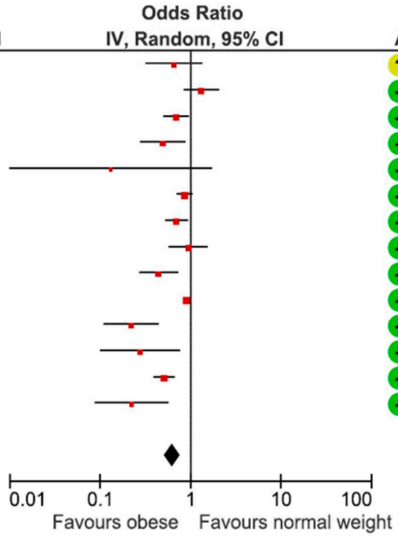

Risk of bias legend
(**A**) Selection Bias
(**B**) Performance Bias
(**C**) Attrition Bias
(**D**) Detection Bias
(**E**) Overall study quality (+ = High quality; ? = Acceptable; - = Low quality)

The risk of bias summary produced from the critical appraisal according to the SIGN cohort appraisal checklist is also displayed for each included study.

Supplementary Figure S8: Forest plot displaying the risk of long-term mortality following MI in underweight (BMI < 18.5 kg/m^2) patients compared to normal weight (BMI 18.5–24.9 kg/m^2) for the studies using unadjusted hazard ratio.

BMI (underweight vs normal weight) and post-MI long-term mortality – unadjusted HRs

Study or Subgroup	log[Hazard Ratio]	SE	Underweight Total	Normal weight Total	Weight	Hazard Ratio IV, Random, 95% CI
Bucholz 2016b	0.5128	0.0155	5678	51896	39.7%	1.67 [1.62, 1.72]
Kim 2019	1.2296	0.1301	2632	5368	22.7%	3.42 [2.65, 4.41]
O'Brien 2014	0.5068	0.0383	1236	11186	37.6%	1.66 [1.54, 1.79]
**Total (95% CI)**			**9546**	**68450**	**100.0%**	**1.96 [1.63, 2.36]**

Heterogeneity: Tau² = 0.02; Chi² = 30.08, df = 2 (P < 0.00001); I² = 93%
Test for overall effect: Z = 7.16 (P < 0.00001)

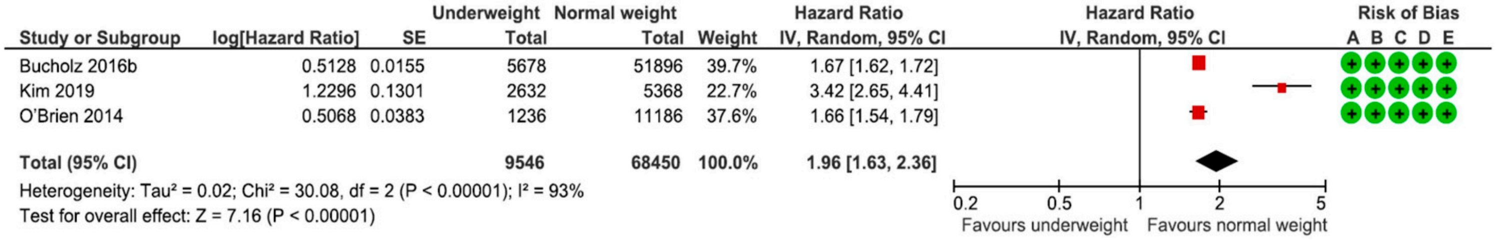

Risk of bias legend
(**A**) Selection Bias
(**B**) Performance Bias
(**C**) Attrition Bias
(**D**) Detection Bias
(**E**) Overall study quality (+ = High quality; ? = Acceptable; - = Low quality)

The risk of bias summary produced from the critical appraisal according to the SIGN cohort appraisal checklist is also displayed for each included study.

Supplementary Figure S9: Forest plot displaying the risk of long-term mortality following MI in underweight (BMI < 18.5 kg/m^2) patients compared to normal weight (BMI 18.5–24.9 kg/m^2)for the studies using unadjusted odds ratio.

BMI (underweight vs normal weight) and post-MI long-term mortality – unadjusted ORs

Study or Subgroup	log[Odds Ratio]	SE	Underweight Total	Normal weight Total	Weight	Odds Ratio IV, Random, 95% CI	Risk of Bias A	B	C	D	E
Aronson 2010	1.4576	0.4055	26	618	16.2%	4.30 [1.94, 9.51]	+	+	+	+	+
Kragelund 2005 (men)	0.9163	0.366	41	1613	19.5%	2.50 [1.22, 5.12]	+	+	+	+	+
Kragelund 2005 (women)	0.9361	0.2442	120	989	38.4%	2.55 [1.58, 4.12]	+	+	+	+	+
Yokoyama 2019	0.5082	0.3119	123	126	25.8%	1.66 [0.90, 3.06]	+	+	+	+	+
**Total (95% CI)**			**310**	**3346**	**100.0%**	**2.48 [1.77, 3.47]**					

Heterogeneity: Tau² = 0.02; Chi² = 3.49, df = 3 (P = 0.32); I² = 14%
Test for overall effect: Z = 5.28 (P < 0.00001)

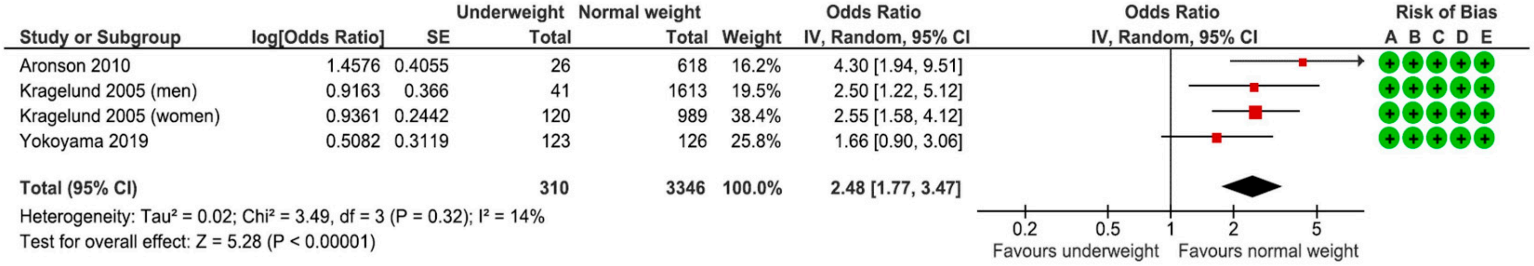

Risk of bias legend
(**A**) Selection Bias
(**B**) Performance Bias
(**C**) Attrition Bias
(**D**) Detection Bias
(**E**) Overall study quality (+ = High quality; ? = Acceptable; - = Low quality)

The risk of bias summary produced from the critical appraisal according to the SIGN cohort appraisal checklist is also displayed for each included study.
